# Is Overweight a Risk Factor for Adverse Events during Removal of Impacted Lower Third Molars?

**DOI:** 10.1155/2014/589856

**Published:** 2014-12-08

**Authors:** Ricardo Wathson Feitosa de Carvalho, Belmiro Cavalcanti do Egito Vasconcelos

**Affiliations:** School of Dentistry, University of Pernambuco, Avenida General Newton Cavalcanti, No. 1650, 54753-220 Camaragibe, PE, Brazil

## Abstract

Being overweight is recognised as a significant risk factor for several morbidities; however, the experience of the dentistry faculties focusing on this population is still low. The aim of the present study was to determine the occurrence of adverse events during removal of impacted lower third molars in overweight patients. A prospective cohort study was carried out involving overweight patients subjected to surgical removal of impacted lower third molar as part of a line of research on third molar surgery. Predictor variables indicative of the occurrence of adverse events during surgery were classified by their demographic, clinical, radiographic, and surgical aspects. Descriptive and bivariate statistics were computed. In total, 140 patients fulfilled the eligibility criteria, and 280 surgeries were performed. Patients' mean age was 25.1 ± 2.2 years, and the proportion of women to men was 3 : 1. Eight different adverse events during surgery were recorded. These events occurred in approximately 29.3% of cases and were significantly associated with predictor variables (*P* < 0.05). Excess weight is recognised as a risk factor for the high rate of adverse events in impacted third molar surgery. The study suggests that overweight patients are highly likely to experience morbidities.

## 1. Introduction

Adverse events are unintended consequences of health care and occur with frequency [1]. A large number of these events could be avoided by using evidence-based practices and the implementation of security measures [2], such as standardisation and simplification of procedures and the recognition of associated factors.

The estimate of possible adverse events is a common dilemma in health care [3]. In oral surgery, the removal of third molars is the most common procedure [4], and as a reflection of the global epidemics of overweight and obesity [5], an increasing number of overweight patients are undergoing third molar surgery, constituting an ongoing challenge for oral and maxillofacial surgeons.

Obesity has long ceased to be just an aesthetic issue. For many surgeons, removing third molars is more difficult and prone to complications when performed in overweight patients; however, there is no evidence in the medical literature (PubMed) of studies regarding conducting third molar surgery in overweight and obese patients so far, disregarding the existence of this portion of the population.

Because of the scarcity of scientific evidence on this issue, the aim of the present study was to determine the occurrence of adverse events during removal of impacted lower third molars in overweight patients. The researchers hypothesise that the occurrence of these events is high in overweight patients. The specific aim was to identify and clarify the variables of interest for such occurrences.

## 2. Material and Methods

### 2.1. Study Design, Location, and Eligibility Criteria

To achieve the research purpose, researchers have designed and implemented a prospective cohort study conducted between January 2011 and December 2012. The study population consisted of overweight patients that came for the evaluation and surgical procedure of impacted lower third molars in the Department of Oral and Maxillofacial Surgery, University of Pernambuco, Brazil. The eligibility criteria were the following: indication for surgery under local anaesthesia and categories I and II of the American Society of Anesthesiology (ASA I and II). The exclusion criteria were age younger than 18 years, absence of second lower molar, body mass index (BMI) less than 18.5 kg/m^2^, BMI greater than 29.9 kg/m^2^, systemic and/or behavioural disorder that rendered local anaesthesia unviable, being pregnant or being lactating women, recent irradiation, cognitive impairment that rendered the comprehension of the study objectives impossible, and the nonacceptance of the methodology. All patients signed terms of informed consent, and the study received approval from the ethics committee of the University of Pernambuco, Brazil (ClinicalTrials.gov Brazil identifier CAAE: 0241.0.097.000-11).

### 2.2. Sample Size

Knowing the proportion of the overweight Brazilian population (50.1%) [6], it was used in the formula for calculating the sample size for a reliable estimate of the population proportion at 90% confidence level (*Z* *α*/2 = 1.645) and estimating the maximum error (*E*) within ±5% (0.05), and the sample was estimated in 280 surgical interventions.

### 2.3. Interpretation and Recording of Predictor Variables

In the preoperative phase, weight and height were measured and recorded to calculate the BMI. The predictor variables were categorised into the following groups: demographic (gender and age), clinical (associated pathologies), and radiographic (level of occlusal plane, available retromolar space, Winter's classification of impaction, number of roots, root curvature, tooth relation with mandibular canal, relation to second molar, crown width, and periodontal space).  Table 1 summarises the predictor variables and their definitions. Predictor variables were recorded by a single examiner. Further data were obtained from digital orthopantomography (panoramic picture). After the initial examination, the patients were randomly sent to two previously calibrated senior surgeons who had no contact with the patients in the preselection phase and were blinded to the previously collected data.

### 2.4. Interpretation and Recording of Adverse Events

*Adverse events* (primary outcome variable) in the present study were defined as* any undesirable, unintentional result affecting the patient at the time of surgery that would not have occurred if the operation had gone as planned, requiring additional management beyond that originally planned by the surgeon* (yes/no) [7]. To avoid subjectivity and imprecision, qualitative terms (secondary outcome variables) such as* small complication* or* large complication* were purposely avoided in the reports. The revised definition suggests that an* adverse event* is not a fixed reality.

### 2.5. Record of Adverse Events

Immediately prior to surgery, the surgeon wrote down the entire surgical plan of the case from incision to suturing. During the procedure, an examiner verified the technical manoeuvres used for the extraction and recorded any intraoperative event that required management beyond that which was originally planned. Surgery time from incision to suturing was also recorded (operative variable).

### 2.6. Surgical Technique

All procedures were carried out in the same surgery unit with the same instruments, high-speed drills (80,000 to 150,000 rpm, conical bit no. 702), and materials. Local anaesthesia was administered (3% lidocaine with noradrenaline at 1 : 50,000) for the regional blocking of the lower alveolar, lingual, and buccal nerves after aspiration. No sedation method was used in the present study. All extractions were carried out with the standardised general method for the surgical removal of impacted third molars described by Farish and Bouloux [8].

### 2.7. Statistical Methods

Descriptive and bivariate statistics were computed, and a model was adjusted to explain each of the predictor variables. A model was first adjusted for each predictor variable considering all independent variables with a level of significance up to 15% (*P* < 0.15). The adjustment of the final model was performed using the backward stepwise procedure, maintaining only those variables with a level of significance up to 5.0% (*P* < 0.05). The Statistical Package for the Social Sciences (SPSS, version 15.0) was used for the statistical calculations.

## 3. Results

In total, 280 surgical interventions were performed in the 140 patients included in the study. Bilateral extractions were performed in all patients, but all interventions were performed on different occasions. The mean patient age was 25.1 ± 2.2 years. The female-to-male gender proportion was 3 : 1 (74.3% and 25.7%, resp.). Most patients had lower third molars with two or more roots (54.3%), and root laceration proved to be frequent among overweight patients (21.4%). The radiolucent periodontal space and absence of pathologies were found in three fourth of the sample, observing an important frequency of dental ankylosis and cases of pericoronitis in overweight patients. The relationship with the mandibular canal was observed in 45.7% of the sample.  Table 2 shows the descriptive statistics of the sample.

Based on Winter and Pell-Gregory classifications, the most frequent tooth position was vertical (62.9%), with high occlusal plane level (74.3%) and sufficient or reduced retromolar space (55.7% and 35.7%, resp.). The crown morphology was not bulbous in more than half of the sample (68.6%). The highest percentage of third molars was in contact with the second molars, a more common condition (58.6%) (Table 2).

**Figure 1:**
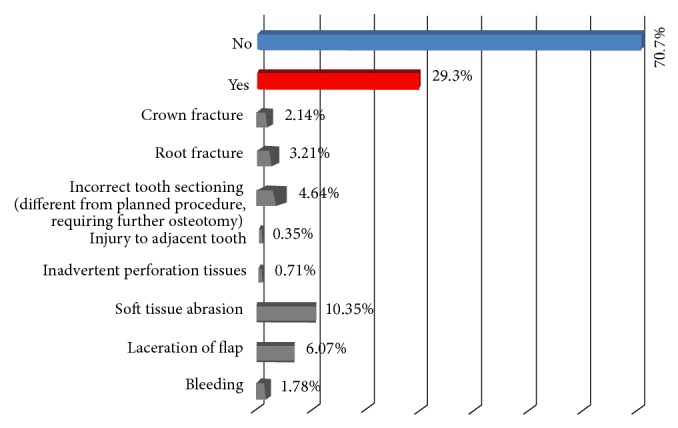
Occurrence of adverse events during the removal of impacted lower third molars in overweight patients.

**Figure 2:**
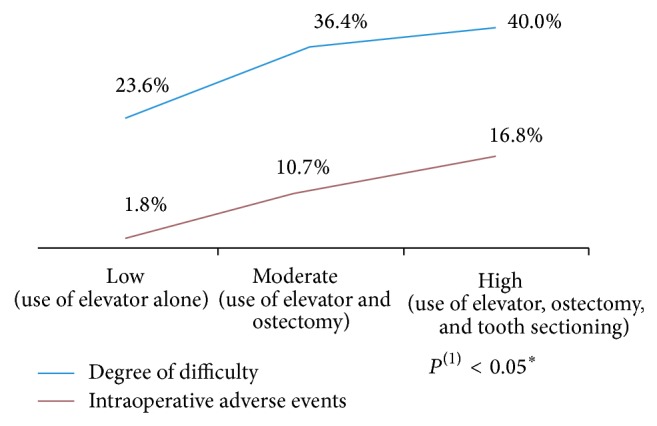
Analysis of the correlation between surgical difficulty and occurrence of intraoperative adverse events.

Eight different intraoperative events required additional management beyond that originally planned and were recorded as adverse events. These events occurred in approximately three of every 10 surgeries (Figure 1). More than half of the procedures performed in this study on overweight patients demanded to perform osteotomy/dental section (76.4%), which required moderate surgical time (15 to 30 minutes) (67.2%). A positive association was found between the degree of difficulty and the occurrence of intraoperative adverse events (Figure 2).

Table 2 displays the bivariate associations between the predictive variables and the occurrence of adverse events.

The odds ratios revealed that the likelihood of adverse events during the surgical removal of an impacted lower third molar in overweight patients was associated with these characteristics: (1) if the patient is male rather than female, (2) if it is classified according to Pell and Gregory as C2 compared with those classified as A1, (3) if the patient has two or more roots in comparison with those with a fused root or germ, (4) if it is associated with injury, (5) if there is a close relationship between the crown and the root of the second molar compared with those without contact with the second molar, and (6) if there is a reduced periodontal space (Table 2).

## 4. Discussion

Overweight contributes significantly to a burden of chronic diseases and disabilities, being considered a public health problem [5, 9]. The pathophysiological changes associated with this disease undertake much of the body's systems, further hindering their treatment, challenging specialists from different health areas.

The impacted third molar surgery has been demonstrated to have a low frequency of complications [10]; however, the results of this study confirm that the approach on overweight patients is also challenging for oral and maxillofacial surgeons, finding a high frequency of intraoperative adverse events (29.3%); overweight patients have high risk of experiencing morbidities during the removal of impacted lower third molars.

Diligence in surgery goes beyond just treating postoperative adverse events and requires enough attention to preoperative details in order to avoid errors and complications [11]. This study shows the existence of predictive variables associated with the occurrence of adverse events in the surgery of impacted lower third molars in overweight patients.

Researchers and surgeons agree that adverse events are more often associated with more difficult extractions [4]. The results of this study show that the highest percentage of surgeries performed in overweight patients demanded to perform osteotomies/dental sections and longer surgical time (31 ± 2.5 minutes), increasing the degree of difficulty and occurrence of intraoperative adverse events.

A recently published study focusing on the occurrence of intraoperative adverse events shows a frequency of 6.8% [7]. The results of this study suggest a frequency of about 29.3% of adverse events during the removal of lower third molars in overweight patients, demonstrating that the occurrence of these events is approximately fourfold. The lack of similar studies involving overweight patients impedes the comparison of data from different centres.

This sample demonstrates that women seek third molar surgery more often than men (3 : 1). However, the results show that the male gender is a determinant predictive factor of intraoperative adverse events, having already been shown in the literature that gender is a risk factor [12]. The authors make this attribution to the fact that men have a thicker mandible, requiring greater manipulation that predisposes to a higher incidence of complications.

Complications are justified and accepted by most surgeons when teeth are associated with pathological processes [13]. Statistical tests revealed significant associations between diseases and complications present in over half of cases associated with caries and fluctuated among other diseases. This pathological condition leads to reduced tooth structural strength, predisposing the occurrence of unwanted fractures that commonly require additional management to the planning, being a common condition in overweight patients, assuming that the prophylactic removal of third molars without pathological associations reduces the risk of intraoperative adverse events in these patients.

The tooth bone depth has been described as the most important indicator for predicting difficulty [14, 15]. Teeth deviation from a vertical alignment was shown to increase the difficulty because of the limited access to the tooth rotational axis, but it was shown not to increase the possibility of adverse events. However, the higher occurrence rate of these events was found depending on the tooth position according to the classification of Pell and Gregory (>C2) [16]. The results also show that although most of the impacted third molars in overweight patients have minor level of inclusion (A1 Vertical), the extractions are performed with a high degree of difficulty by requiring greater manipulation. The authors believe that this result is a reflection of the reduced visibility of the surgical field often predisposing to unwanted tooth sectioning and requiring additional management with the planning.

The atretic periodontal space proved to be a frequent condition in overweight patients (5.75%), leading us to believe that it can be attributed to larger and more frequent chewing activity, which leads to a constant load in this space, leaving it atretic, proving to be another significant indicator of intraoperative adverse events (*P* ^(1)^ = 0.025^*^). The narrowing or lack of space because of the tooth fusion to the alveolus (tooth ankylosis) prevents or hinders an indispensable step in the surgical removal of impacted third molars: periodontal space dilation through tooth dislocation before the action at the lever point [8]. With this expansion of the periodontal space partially performed or not performed, there is an increased risk of the occurrence of adverse events, which is more likely with the presence of two or more roots (*P* ^(1)^ < 0.001^*^). To minimise the risk, this condition requires a longer dislocation and the use of high-speed rotary instruments to reduce or eliminate fusion areas before elevator application.

The evidence shows that the relationship between third molar's crow and the second molar is a significant predictive variable for the occurrence of adverse events in surgery of impacted lower third molars in overweight patients (*P* ^(1)^ < 0.001^*^). The closeness between the second and third molars represents an additional risk [8]. A close relationship between these teeth reduces the space between the distal surface of the second molar and third molar mesial surface, preventing the access to the lever point, which is a key point for removing impacted third molars [17]. This lack of access to the tip of the elevator tends to make the surgeon apply a greater force to reach this point, thus predisposing the patient to local adverse events such as flap laceration, soft tissue abrasion, inadvertent perforation of soft tissues, injury to adjacent tooth, root fracture, and crown fracture.

## 5. Conclusion

This study minimises the limited experience of dentistry faculties, focusing on this population, showing that overweight patients are highly susceptible to experience adverse events in the removal of impacted lower third molars, warning the students and dentists with no experience in caring for overweight patients. The understanding and the detection of predictor variables may be useful for students and professionals who perform lower third molar surgery in overweight patients to break the current medical attitude in treating the complications of being overweight and obese.

## Figures and Tables

**Table 1:** Predictor variables evaluated.

Groups of predictor variables	Variable/definition	Classification
Body weight	**Body mass index (BMI)** (weight [kg] divided by height squared [m^2^])	1: 25.0–29.9 (overweight)

Demographic	Gender	1: Female
		2: Male
	Age	1: <25 years
		2: ≥25 years

Clinical	**Associated pathologies** (condition associated to the 3rd molar)	1: None
		2: Pericoronitis
		3: Caries
		4: Bone resorption

Radiographic	**Level of occlusal plane**—Pell and Gregory (occlusal plane of the 3rd molar in relation to the 2nd molar)	1: High—larger part of crown of the 3rd molar above or on the same level as the 2nd molar
		2: Medium—larger part of crown of the 3rd molar between the occlusal plane and the cementoenamel junction of the 2nd molar
		3: Low—crown of the 3rd molar completely below the cementoenamel junction of the 2nd molar
	**Available retromolar space**—Pell and Gregory (distance between distal-most point of the 2nd molar crown and the anterior-most point of the ascending ramus)	1: (A) Sufficient—space greater than or equal to the mesiodistal distance of the 3rd molar
		2: (B) Reduced—space greater than half and less than the mesiodistal distance of the 3rd molar
		3: (C) Insufficient—space less than half of the mesiodistal distance of the 3rd molar
	**Impaction angle** (Winter), measured in degrees (angle between the crossing of the long axis of the 3rd molar and the occlusal plane)	1: Horizontal 0° to 30°
		2: Mesioangular 31° to 60°
		3: Vertical 61° to 90°
		4: Distoangular > 90°
	**Number of roots**	1: One fused root
		2: ≥2 roots
		3: Tooth germ
	**Root curvature** (angle between the long axis of the crown and the root of the 3rd molar)	1: Nondilacerated < 10°
		2: Dilacerated > 10°
	**Tooth relation with mandibular canal** (distance [mm] from the root apex to the cortex of mandibular canal)	1: Negative—apex with no contact with the cortex of the mandibular canal
		2: Positive—apex in contact with the cortex of the mandibular canal
	**Relation with the 2nd molar **(relation of the 3rd molar crown with the 2nd molar)	1: No contact
		2: Contact with the crown alone
		3: Contact with the crown and the root
		4: Contact with the root alone
	**Crown width** (mesiodistal distance of the 3rd molar crown compared with the 2nd molar)	1: Nonbulbous (equal to or less than that of the 2nd molar)
		2: Bulbous (greater than that of the 2nd molar)
	**Periodontal space** (status of space between the root of the 3rd molar and the alveolar cortex)	1: Radiolucent (fully radiolucent space)
		2: Mixed (radiolucent and radioopaque)
		3: Radioopaque (totally radiopaque space)

**Table 2:** Distribution of patients according to predictive variables and correlation of variables and occurrence of intraoperative adverse events.

Variable	Overweight patients			Occurrence of adverse events			
	Classification	n	%	Yes		P value	OR (IC to 95%)
				n	%		
Age	<25 years	132	47.1	34	25.8	*P* ^(1)^ = 0.221	1.00
	≥25 years	148	52.9	48	32.4		1.38 (0.82 to 2.33)

Gender	Female	208	74.3	50	24.0	*P* ^(1)^ = 0.001^*^	2.53 (1.44 to 4.44)
	Male	72	25.7	32	44.4		1.00

Associated pathologies	None	212	75.7	54	25.5	*P* ^(2)^ < 0.001^*^	∗∗
	Pericoronitis	40	14.3	14	35.0		∗∗
	Caries	16	5.7	13	81.3		∗∗
	Bone resorption	12	4.3	1	8.3		∗∗

Level of occlusal plane—Pell and Gregory	High	208	74.3	62	29.8	*P* ^(1)^ = 0.021^*^	∗∗
	Medium	56	20.0	20	35.7		∗∗
	Low	16	5.7	∗∗	∗∗		∗∗

Available retromolar space—Pell and Gregory	Sufficient	156	55.7	34	21.8	*P* ^(1)^ = 0.001^*^	1.00
	Reduced	100	35.7	34	34.0		1.85 (1.05 to 3.24)
	Insufficient	24	8.6	14	58.3		5.02 (2.05 to 12.31)

Impaction angle (Winter)	Horizontal	28	10.0	6	21.4	*P* ^(1)^ = 0.605	1.00
	Mesioangular	76	27.1	22	28.9		1.62 (0.62 to 4.23)
	Vertical	176	62.9	54	30,7		1.49 (0.53 to 4.18)
	Distoangular	—	—	∗∗	∗∗		

Number of roots	One fused root	112	40.0	22	19.6	*P* ^(1)^ < 0.001^*^	∗∗
	≥2 roots	152	54.3	60	39.5		∗∗
	Tooth germ	16	5.7	∗∗	∗∗		∗∗

Root curvature	No	220	78.6	66	30.0	*P* ^(1)^ = 0.615	1.00
	Yes	60	21.4	16	26.7		1.18 (0.62 to 2.24)

Tooth relation with mandibular canal	No	152	54.3	44	28.9	*P* ^(1)^ = 0.892	1.00
	Yes	128	45.7	38	29.7		1.04 (0.62 to 1.74)

Relation with the 2nd molar	None	116	41.4	30	23.4	*P* ^(1)^ < 0.001^*^	1.00
	Crown alone	128	45.7	6	30.0		1.40 (0.50 to 3.96)
	Crown/root	20	7.1	12	75.0		9.80 (2.94 to 32.64)
	Root alone	16	5.7	34	29.3		1.35 (0.77 to 2.40)

Crown width	Nonbulbous	192	68.6	52	27.1	*P* ^(1)^ = 0.232	1.39 (0.81 to 2.40)
	Bulbous	88	31.4	30	34.1		1.00

Periodontal space	Radiolucent	200	71.4	49	24.5	*P* ^(1)^ = 0.025^*^	1.00
	Mixed	64	22.9	24	37.5		2.27 (1.21 to 4.27)
	Radioopaque	16	5.7	9	56.3		4.45 (1.50 to 13.44)

**Total**		**280**	**100.0**	**82**	**29.3**		

^*^Significant association at 5.0%, (—) Undetermined because of sample size, ^(1)^Pearson's chi-square test, ^(2)^Fisher's exact test.
